# Tai Chi for the elderly patients with COVID-19 in recovery period: A protocol for systematic review and meta-analysis

**DOI:** 10.1097/MD.0000000000024111

**Published:** 2021-01-22

**Authors:** Xiangyu Zhu, Ziyu Luo, Ying Chen, Lina Wang, Wenxin Chi, Lu lian Jiang, Ke Liu, Liping Zhao, Yu Zhang, Haibo Zhang

**Affiliations:** a School of Acupuncture, Moxibustion and Tuina; b Dongzhimen Hospital, Beijing University of Chinese Medicine; c Laboratory of Statistics and Measurement, Beijing Sport University, Beijing, China.

**Keywords:** coronavirus disease 2019, elderly patients, meta-analysis, systematic review, Tai Chi

## Abstract

**Background:**

The coronavirus disease 2019 (COVID-19) outbreak has caused a great impact in many countries. Older people are more susceptible to the virus than other people. As a good health exercise suitable for the elderly, Tai Chi has a positive impact on heart function, blood pressure, lung function, immunity, etc. It can enhance cardiopulmonary function, increase the elasticity of blood vessels, and improve the body's self-regulation function. For the elder patients with COVID-19, Tai Chi has outstanding merits.

**Methods:**

We will search PubMed, EMBASE, MEDLINE, the Cochrane Library, Chinese National Knowledge Infrastructure, Chinese Biomedical Literature Database, Chinese Science and Technology Periodical Database, Wanfang Database, Clinical Trials and Chinese Clinical Trial Registry. The complete process will include study selection, data extraction, risk of bias assessment and meta-analyses. Endnote X9.3 will be used to manage data screening. The statistical analysis will be completed by Stata/SE 15.1 software.

**Results:**

This proposed study will evaluate the effectiveness and safety of Tai Chi for the improvement of psychological pressure, cardiopulmonary function, and immunity in elderly COVID-19 patients during the recovery period.

**Conclusion:**

The conclusion of this study will provide evidence to prove the safety and effectiveness of Tai Chi on elderly COVID-19 patients during the recovery period.

**Ethics and dissemination:**

This protocol will not evaluate individual patient information or infringe patient rights and therefore does not require ethical approval.

**Registration:**

PEROSPERO CRD42020220128

## 1 Introduction

The type of pneumonia caused by the 2019 novel coronavirus disease (COVID-19) is a highly infectious disease, and the ongoing outbreak has been declared by WHO as a global public health emergency.^[1,2]^

The studies reveal that age is by far the strongest predictor of an infected person's risk of dying—a metric known as the infection fatality ratio (IFR), which is the proportion of people infected with the virus, including those who didn’t get tested or show symptoms, who will die as a result.^[3]^ For every 1,000 people infected with the coronavirus who are under the age of 50, almost none will die. For people in their fifties and early sixties, about five will die. The risk then climbs steeply as the years accrue. For every 1,000 people in their mid-seventies or older who are infected, around 116 will die. These are the stark statistics obtained by some of the first detailed studies into the mortality risk for COVID-19.^[4]^

The World Health Organization (WHO) reports that by far, the largest public mental health impact has been in the form of stress and anxiety and predicts a rise in depression, suicide, and substance use in the coming days.^[5]^ According to a recently developed emotional epidemic curve without adequate mitigation measures, countries will experience the first peak of negative mental health consequences, which corresponds to the peak in COVID-19 cases.^[6]^ A meta-analysis of survivors of the SEVERE acute respiratory syndrome (SARS) and the Middle East Respiratory Syndrome (MERS) coronavirus showed: impaired diffusing capacity for carbon monoxide, reduced exercise capacity, prevalences of post-traumatic stress disorder, depression, and anxiety are common symptoms 6 months after discharge.

A study found that some patients were depressed, eating less, insomnia, and not willing to communicate with others in Fangcang hospital, Hubei province, China. This mentality came from the worries of relatives, the indeterminacy of the illness state, the death of wardmate, and increased the psychological pressure of patients.^[7]^

In recent years, several trials have confirmed that Tai Chi can improves patient anxiety of old patients.^[8,9]^ According to a study conducted by Kim, Tai Chi may lower sympathetic tone and increase parasympathetic tone, which may result in changes in the autonomic nervous system.^[10]^ In addition, a randomized controlled study conducted by Tsai et al suggested that a 12-week period of Tai Chi exercise reduces blood pressure, as well as lipid levels, and improves patient anxiety.^[11,12]^

Clinical and epidemiological features of COVID-19 demonstrate that the infection can cause clusters of severe respiratory illness, leading to intensive care unit (ICU) admission and high mortality.^[13]^ SARS and MERS exhibit some similarities to COVID-19, but COVID-19 can cause a wider range of symptoms associated with many-body systems, such as the heart, kidneys, and nervous system, and may have a greater impact on the quality of life (QoL).^[14–16]^ In addition, prolonged hospitalization or bedridden treatment for COVID-19 survivors can lead to a sustained reduction in physical activity, which can lead to increased pain and deterioration of joint function.^[17]^ To sum up, COVID-19 will reduce the body function, reduce the exercise ability, and have an unhealthy impact on psychological health.

There has been increasing interest in Tai Chi as an exercise treatment method for various diseases.^[18–25]^ Researchers from the Institute of Integral Qigong and Tai Chi, Arizona State University, and the University of North Carolina analyzed 77 articles.^[26]^ Current research suggests that the strongest and most consistent evidence of health benefits for Tai Chi is for bone health, cardiopulmonary fitness, immunity, and factors associated with preventing falls, quality of life, and self-efficacy.^[27]^ The reviewers concluded that the evidence is sufficient to suggest that tai chi is a viable alternative to conventional forms of exercise.^[28,29]^

Therefore, we will investigate the effect of Tai Chi on the mentality, cardiopulmonary function, and immunity of the elderly patients with COVID-19 in recovery period in a systematic review and meta-analysis.

## 2 Methods

### 2.1 Registration

The study protocol has been registered on international prospective register of systematic review (PROSPERO registration number: CRD 42020220128). The procedure of this protocol will be conducted according to the Preferred Reporting Item for Systematic Review and Meta-analysis Protocols (PRISMA-P) guidance.^[35]^

### 2.2 Inclusion and exclusion criteria

#### 2.2.1 Type of study

Randomized controlled trials (RCTs) about Tai Chi for COVID-19 in recovery period will be included. Non-RCTs, quasi-RCTs, case series, reviews, animal studies and any study with a sample size of less than ten participants will be excluded.

#### 2.2.2 Type of participant

Elderly COVID-19 patients (over 60 years old) who have been clearly diagnosed and now in recovery period, regardless of sex, age, race or educational and economic status, will be included in the review.

#### 2.2.3 Type of interventions

Interventions can be any type of Tai Chi, such as Yang's Tai Chi, Chen's Tai Chi and other types of Tai Chi. Multiple control measures will be included, such as blank, placebo, usual or standard care, health education, psychosocial therapy, drug therapy. Any comparisons between a combined therapy of Tai Chi exercises and other interventions with a therapy of solely using other interventions are also included. All the frequencies, durations, and types of Tai Chi exercises will be considered.

#### 2.2.4 Type of outcome measures

Outcome indicators include effectiveness indicators and safety indicators. Effectiveness indicators include primary outcome indicators and secondary outcome indicators. The primary outcome indicators are 1-second forced expiratory volume (FEV1), 1-second forced vital capacity (FEV1/FVC), blood oxygen saturation, total white blood cell count, the content of IgG, IgM, IgA, C3, and C4 in serum, blood oxygen saturation, the Patient Health Questionnaire (PHQ-9), the Hearth Hope Index (H H Index). The secondary outcome indicators are the disappearance time of main symptoms (including fever, asthenia, cough disappearance rate, and temperature recovery time), negative COVID-19 results rates on two consecutive occasions (not on the same day), CT image improvement, average hospitalization time, occurrence rate of common type to severe form, clinical cure rate, and mortality. Safety is referred to the incidence of adverse events (bleeding, pain, hematoma, syncope, etc.).

### 2.3 Search strategy

The following electronic bibliographic databases will be searched to identify relevant studies: PubMed, EMBASE, MEDLINE, the Cochrane Library, Chinese National Knowledge Infrastructure (CNKI), Chinese Biomedical Literature Database (CBM), Chinese Science and Technology Periodical Database (VIP), Wanfang Database, Clinical Trials and Chinese Clinical Trial Registry. A combination of subject words and free text words will be applied in the searches. The references of systematic reviews and literature included will also be checked. The search strategies for selecting the fields of title, abstract or keyword will be adjusted according to different characteristics of databases. The language is limited to Chinese and English. The search terms are shown in Table 1.

**Table 1:** Search strategy of PubMed.

Search	Query
#1	“COVID-19”[Mesh Terms]
#2	“COVID19”[Title/Abstract] OR “SARS-CoV-2”[Title/Abstract] OR “2019-nCoV”[Title/Abstract] OR “2019 nCoV”[Title/Abstract] OR “2019nCoV”[Title/Abstract] OR “2019 novel coronavirus”[Title/Abstract] OR “coronavirus disease 2019”[Title/Abstract] OR“coronavirus disease-19”[Title/Abstract] OR “Wuhan seafood market pneumonia virus”[Title/Abstract] OR “Wuhan coronavirus”[Title/Abstract]
#3	#1 OR #2
#4	“Tai Ji”[Mesh]
#5	“Tai-ji”[Title/Abstract] OR “Tai Chi”[Title/Abstract] OR “Chi, Tai”[Title/Abstract] OR “Tai Ji Quan” [Title/Abstract] OR “Ji Quan,Tai”[Title/Abstract] OR “Quan, Tai Ji”[Title/Abstract] OR “Taiji”[Title/Abstract] OR “Taijiquan”[Title/Abstract] OR “T’ai Chi”[Title/Abstract] OR “Tai Chi” Chuan[Title/Abstract]
#6	#4 OR #5
#7	“randomized controlled trial”[Publication Type] OR “randomized”[Title/Abstract] OR “placebo”[Title/Abstract]
#8	#3 AND #6 AND #7

### 2.4 Study selection

**Figure 1:**
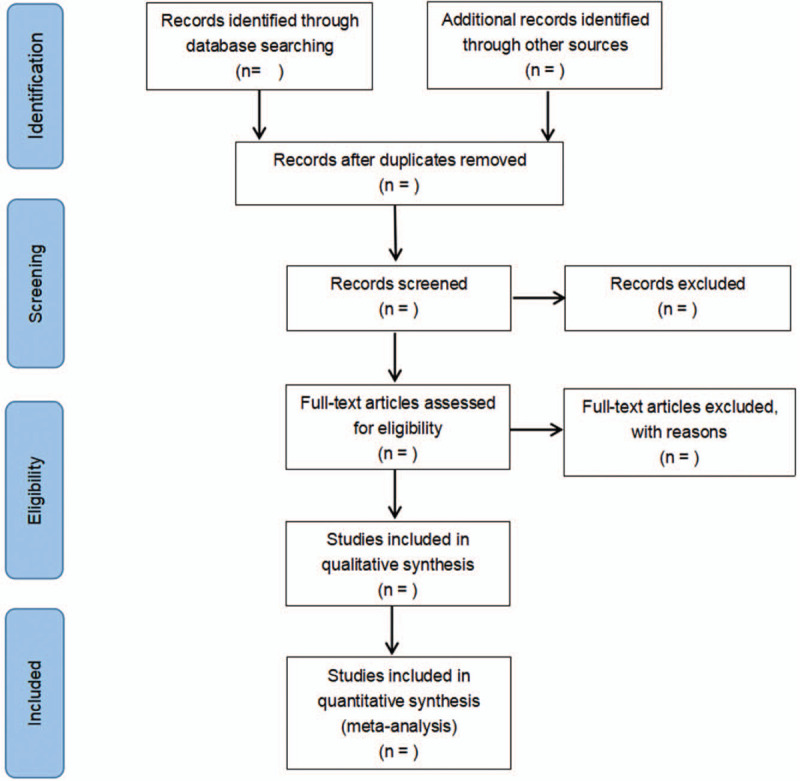
It is the PRISMA flow chart presenting details of the selection process.

The literature will be retrieved according to the retrieval strategy, then imported them into the literature management software. Endnote version 9.3 (The Thomson Corporation Corp, Stanford, CT) will be used to manage data screening. The research on duplicate titles was deleted, and obviously irrelevant literature was excluded by reading titles and abstracts. The above steps were performed independently by two researchers. Any disagreements will be resolved by discussion with a third researcher. The researchers will record all studies that do not meet the inclusion criteria and provide the rationale for their exclusion. Details of the selection process will be presented in the PRISMA flow chart. (Fig. 1)

### 2.5 Data extraction

Extract data from selected studies, which include general information, reference (name of the leading author and year of publication, and study design), participant characteristic, intervention, methods, control, training frequency and length, outcomes measured, results, adverse reactions. The above steps were performed independently by two researchers. Any disagreements will be resolved by discussion with a third researcher. If required information is not reported, we will try our best to contact the corresponding authors of the studies through email to deal with missing data. And the study will be further excluded without adequate information.

### 2.6 Risk of bias assessment

Two authors will assess methodological quality of included studies separately by the Cochrane collaboration's risk of bias tool.

We will consider the following:

(1) random sequence generation (selection bias)
(2) allocation concealment (selection bias)
(3) blinding of participants and personnel (performance bias)
(4) blinding of outcome assessment (detection bias)
(5) incomplete outcome data (attrition bias)
(6) selective reporting (reporting bias)
(7) other sources of bias (other bias).

The bias risk in each aspect will be assessed and divided into 3 levels: low risk, high risk, and unclear risk.

### 2.7 Statistical analysis

#### 2.7.1 Strategy for data synthesis

Stata/SE version 15.1 (STATA Crop., College Station, TX) will be used to conduct this meta-analysis. The groups included in synthesis must meet our inclusion criteria, using a recognized quality of life scale. The data results will be calculated as the mean difference (MD) or standardized mean difference (SMD) with corresponding 95% confidence intervals (CIs). Heterogeneity will be assessed using the Q test (with *P* < .1 considered to represent significant statistical heterogeneity), and the I^2^ statistic (with I^2^ > 50% considered to be indicative of substantial heterogeneity). If necessary, meta-regression, subgroup, and sensitivity analyses will also be performed to analyze the source of any heterogeneity. Data synthesis calculated using a random-effects or fixed-effects model. We will clearly describe which studies were included and how they have been synthesized as described. We will be transparent about the metric being used.

#### 2.7.2 Analysis of subgroups or subsets

Subgroup analysis will be performed to explain heterogeneity if necessary.

#### 2.7.3 Sensitivity analysis

Different levels of the methodological quality of trails may tend to affect the overall effects. If the Q test and the I^2^ statistic show significant statistical heterogeneity, sensitivity analyses we will conduct sensitivity analysis. Sensitivity analysis is conducted by excluding studies one by one, so that we can determine the source of heterogeneity.

#### 2.7.4 Publication bias

The publication bias will be evaluated by funnel plots by determining whether there are 10 or more studies with the same outcome. In the case of asymmetric funnel plot, subgroup analysis or sensitivity analysis will be performed to investigate possible causes.

#### 2.7.5 Quality of evidence

We will use the Grading of Recommendations Assessment, Development, and Evaluation guidelines for the assessment of the strength of evidence for each outcome. The result will be categorised as high, moderate, low and very low certainty of evidence.

### 2.8 Ethics and dissemination

This systematic review will not require ethical approval because there are no data used in our study that are linked to individual patient data.

## 3 Results

This proposed study will evaluate the effectiveness and safety of Tai Chi for the improvement of psychological pressure, cardiopulmonary function, and immunity in elderly COVID-19 patients during the recovery period.

## 4 Discussion

Tai Chi is a method of moving and concentrating energy that can be done at home and in the hospital of patient friends. It combines to facilitate re-establishing optimal communication among internal organs and build Chi, vital life energy, on an individual level. Taiji can effectively relieve the psychological pressure of patients, enhance their psychological resilience level, improve their level of hope, and promote patients to face the disease and treatment with a positive attitude, which plays a positive role in the prevention and control of the epidemic situation. A study indicated that older adults with moderate sleep complaints can improve self-rated sleep quality through a six-month low to moderate intensity Tai Chi program. Tai Chi appears to be effective as a non-pharmacological approach to sleep enhancement for sleep-disturbed elderly individuals.^[30]^

Taijiquan exercise mainly uses abdominal breathing, which requires deep, long, gentle and smooth breathing, which is consistent with the principle of lung function exercise, that is, the tension and pressure of chest breathing are transferred to the abdomen, so that patients can inhale more oxygen, increase the utilization rate of oxygen, and improve the symptoms of hypoxia. The improvement of symptoms in turn enhances the patients’ confidence in the recovery of the disease, improves the level of hope of the patients, so as to better enable the patients to cope with the disease.

Practicing Tai Chi regularly may reduce the decline of cardio respiratory function in older individuals. A 6-week Tai Chi program indicated significant differences in systolic and diastolic blood pressure for hypertensive patients.^[31]^ One study also showed that Tai Chi exercise training could decrease blood pressure, results in favorable lipid profile changes, and improves subjects’ anxiety status.^[32]^ Older Tai Chi practitioners indicated a higher level of microcirculatory function during exercise than did their sedentary counterparts.^[33]^ Another study indicated the benefit of Tai Chi on blood pressure.^[34]^

Exercise therapy is an active self-healing method, which benefits associated with improved overall physical and mental health, and physical functioning. It may be associated with minimal side effects as compared to drug and surgical interventions.^[35]^ Tai Chi is a traditional physical and mental training. The movements of Tai Chi are lively, continuous and oscillating, which accords with the psychological and physiological characteristics of the elderly.^[36]^ Studies have found that Tai Chi has many positive effects on the health of the elderly, and it is a very suitable exercise for the elderly.^[37,38]^ In the fight against virus infection, it is particularly important to improve the activity of autoimmunity, reduce the body's susceptibility to infectious diseases and strengthen its resistance. Tai Chi exercise can enhance the secretion of erythropoietin and leukocyte stimulating factor in the body, leading to physiological adaptation changes in the blood system, so as to enhance the blood function, especially the immune function. Therefore, Tai Chi has its outstanding advantages in the face of COVID-19.

At present, there is no systematic review of the effects of Tai Chi on the psychological pressure, cardiopulmonary function, and immunity in convalescent elderly patients with COVID-19. It is hoped that this meta-analysis can provide a convincing scientific basis and guide clinical practice. Nonetheless, the lack of sufficient RCTs may be a limitation for this meta-analysis.

## 5 Conclusion

The conclusion of this study will provide evidence to prove the safety and effectiveness of Tai Chi on elderly COVID-19 patients during the recovery period.

## Author contributions

**Conceptualization:** Haibo Zhang.

**Data curation:** Ziyu Luo, Lulian Jiang.

**Formal analysis:** Ying Chen.

**Methodology:** Lina Wang.

**Software:** Wenxin Chi.

**Supervision:** Ke Liu.

**Validation:** Liping Zhao.

**Visualization:** Yu Zhang.

**Writing – original draft:** Xiangyu Zhu.

**Writing – review & editing:** Xiangyu Zhu.
